# The Canadian Healthy Life Trajectories Initiative (HeLTI) Trial: a study protocol for monitoring fidelity of a preconception-lifestyle behaviour intervention

**DOI:** 10.1186/s13063-023-07271-7

**Published:** 2023-04-07

**Authors:** Cindy-Lee Dennis, Flavia Marini, Alessandra Prioreschi, Justine Dol, Catherine Birken, Rhonda C. Bell

**Affiliations:** 1 Lawrence S. Bloomberg Faculty of Nursing, University of Toronto, 155 College Street, Toronto, ON M5T 1P8 Canada; 2 Li Ka Shing Knowledge Institute, St. Michael’s Hospital, Toronto, Canada; 3 Department of Psychiatry, University of Toronto, Toronto, Canada; 4 SAMRC Developmental Pathways for Health Research Unit, Department of Paediatrics, School of Clinical Medicine, University of the Witwatersrand, Johannesburg, South Africa; 5 Faculty of Medicine, University of Toronto, Toronto, Canada; 6 Hospital for Sick Children, Toronto, Canada; 7 Department of Agricultural, Food and Nutritional Sciences, University of Alberta, Edmonton, Canada

**Keywords:** Intervention, Fidelity, Public health, eHealth, Preventative medicine, Randomized controlled trial

## Abstract

**Background:**

In evaluating technology-based behaviour change interventions, it is increasingly important to have a monitoring plan for intervention fidelity. It is important to maintain intervention fidelity to ensure that the theory-based intervention that is being tested is what causes the observed changes, particularly for eHealth behaviour change interventions. In this protocol, we outline the intervention fidelity and monitoring protocol for Healthy Life Trajectory Initiative (HeLTI) Canada, a randomized controlled trial evaluating the effect of a preconception-early childhood technology-based intervention delivered by public health nurses among pregnancy-planning women and their partners to optimize child growth and development.

**Methods:**

The HeLTI Canada fidelity protocol is based on the National Institutes of Health Behaviour Change Consortium (NIH BCC) Treatment Fidelity Framework, outlining the following components of intervention fidelity: study design, provider training, intervention delivery, intervention receipt, and intervention enactment. The intervention fidelity components and associated monitoring strategies were developed to align with the HeLTI Canada approach. Strategies for intervention fidelity monitoring include a pre-post written evaluation of training, standardization of provider training, use and monitoring of activity logs, and intervention session checklists. Possible challenges to intervention fidelity include provider turnover due to the length of the trial and lack of ability to directly monitor participant behaviour change in real-life settings. Details about intervention fidelity monitoring are provided in detail. The study launched in January 2021 and is currently recruiting.

**Discussion:**

Using the NIH BCC Treatment Fidelity Framework, HeLTI Canada has a robust framework for monitoring and reporting intervention fidelity to improve intervention validity, ability to assess intervention effectiveness, and transparency.

**Trial registration:**

ISRCN ISRCTN13308752. Registered on February 29, 2019.

**Supplementary Information:**

The online version contains supplementary material available at 10.1186/s13063-023-07271-7.

## Introduction

Over the past 25 years, there has been rapid growth in digital and mobile health technology, facilitating a rise in technology-based interventions targeting change in health behaviours, such as diet, exercise, and sleep [1]. Technology-based interventions can offer advantages over traditional, in-person interventions, such as increased accessibility and convenience for participants, reduced cost, and the ability to tailor based on individual needs. However, they can also present challenges such as a lack of face-to-face intervention, lower uptake and adoption, and technological difficulties.

Due to the unique implementation factors in technology-based behavioural interventions, intervention fidelity monitoring is of particular importance. Intervention fidelity refers to the methodological strategies and practices used to monitor, enhance, and evaluate the reliability and validity of behavioural interventions [2]. It is important to maintain intervention fidelity to ensure that the theory-based intervention that is being tested is what causes the observed changes.

Despite the importance of fidelity in behavioural interventions, many behavioural trials do not report intervention fidelity protocols or outcomes [3], although existing protocols are available as guidance [4, 5]. A systematic review found that less than 5% of studies reported any treatment fidelity data [6]. This paucity of information on intervention fidelity reporting and fidelity protocol development contributes to the difficulty in the replication and translation of results into practice [2]. Therefore, the purpose of this paper is to describe the intervention fidelity protocol adopted by Healthy Life Trajectory Initiative (HeLTI) Canada, a randomized controlled trial evaluating a preconception-early childhood telephone-based intervention with tailored eHealth resources for women and their partners to optimize growth and development among children in Canada [7]. The protocol was developed using the National Institutes of Health (NIH) Treatment Fidelity Framework, designed by the Behaviour Change Consortium (BCC) [2].

## Methods

### HeLTI Canada overview

The Healthy Life Trajectories Initiative (HeLTI) was developed in partnership with funders from Canada, India, China, and South Africa and in collaboration with the World Health Organization to address the increasing burden of non-communicable diseases (NCDs)—including obesity, diabetes, cardiovascular disease, and poor mental health—around the world. There are four separate but harmonized intervention studies implemented in Soweto, Mysore, Shanghai, and all across Canada. All projects are focused on developing evidence-based interventions that span from preconception across pregnancy and into the postnatal period with the goal of improving maternal, infant and child health, and ultimately the life-long well-being of the child.

The HeLTI Canada study will recruit 5230 women planning a pregnancy within the next 3 years, their partners (if applicable), and their child aged 3 to 12 months (if applicable) across Canada to evaluate whether the 4-phase ‘preconception to early childhood’ life course intervention (preconception, pregnancy, infancy, and early childhood) can, among children born in the trial, at age 5 years: (1) reduce overweight and obese status, (2) reduce zBody Mass Index (zBMI) and improve zBMI trajectories, (3) reduce adiposity, (4) improve cardiometabolic risk factors, (5) enhance development and school readiness, and (6) improve health behaviours including nutrition, physical activity, screen time, and sleep [7]. It will also examine the impact of the intervention on parental outcomes across time. Ethics approval was obtained by St. Michael’s Hospital (Research Ethics Board [REB]# 18–309) and the University of Alberta (REB# 000,092,107).

The HeLTI Canada intervention, with its foundation on public health and primary care platforms and eHealth technologies, takes a ‘cumulative impact’ approach designed to improve health behaviours and reduce modifiable risk factors that influence child obesity. The intervention starts before conception and continues through to early childhood. It is evidence-based, professionally facilitated, proactive, individualized, multifaceted, and sex- and gender-specific, and it builds on existing research and clinical resources while recognizing the growing trend of e-Health [8]. Local public health nurses participate in providing the intervention to ensure it is tailored to local circumstances. Two core strategies will be used throughout the 4 phases: (1) telephone-based collaborative care provided by a HeLTI nurse and (2) HeLTI Canada website with individualized webpage and eHealth resources based on participant needs. Telephone contacts will last 15 to 45 min, based on individual needs. This monitoring will facilitate the early detection of risks that can be targeted. Evidence-based eHealth resources selected by HeLTI Canada co-investigators and experts for targeting health behaviours (e.g. nutrition, physical activity, sleep) and health risks (e.g. depression, smoking cessation, parenting stress) will be easily accessible on a mobile device, tablet, or computer and will enable us to provide innovative and engaging support to participants with diverse health issues.

### Intervention fidelity protocol

The NIH Treatment Fidelity Framework, designed by the BCC [2], has been used to explore the quality of fidelity reporting in health behaviour fields and across behaviour change research. The NIH Treatment Fidelity Framework proposes that the five domains of intervention fidelity listed below are necessary when reporting the results of behavioural intervention trials: (1) study design (e.g. how an intervention was intended to be delivered and includes theoretical frameworks, intended dose, intended content and intended qualifications of intervention providers), (2) intervention provider training (e.g. what specific methods will be used to train providers and maintain provider skills throughout the intervention, (3) intervention delivery (e.g. how well the providers adhere to the intended intervention and includes information about actual dose and content delivered, (4) intervention receipt (e.g. how well the intervention addresses participants’ comprehension of and ability to use learned skills during intervention the intervention period), and (5) intervention enactment (e.g. participants’ ability to use these skills and implement their plans outside of formal intervention sessions).

The following sections outline the five areas of fidelity and the corresponding protocols developed for the HeLTI Canada study (May 2022, version 1). In addition to the development of intervention fidelity protocols, a fidelity monitoring plan (Table 1) was also developed to ensure adherence to the fidelity protocols. Because fidelity strategies are only effective if rigorously implemented, the monitoring plan is intended to ensure the fidelity strategies developed are consistently executed throughout the duration of the study. We used the Standard Protocol Items: Recommendations for Interventional Trials (SPIRIT) reporting guidelines [9] and is outlined in Table 2.

**Table 1:** Overview of HeLTI Canada fidelity monitoring plan

Data source	Monitoring frequency	Areas of fidelity				
		**Study design**	**Provider training**	**Treatment delivery**	**Treatment receipt**	**Treatment enactment**
Discussion board	Daily	X	X	X	X	X
Telephone support 24 h	Daily	X	X	X	X	X
Intervention frequency report	Bi-weekly	X		X		
Intervention logs auditing	Bi-monthly	X		X		
Intervention group sessions	Monthly	X	X	X		
HeLTI Canada App resource bank	Annually			X	X	X
Participant log-in time in HeLTI Canada App	Bi-monthly	X			X	X
Intervention manual review	Bi-annually	X		X	X	
Nurses exit interview	–	X	X			
Participant satisfaction review	3 months after the beginning of each phase			X	X	X
Follow-up training	As needed	X	X	X		
Healthy conversation skills refresher training	Annually	X	X	X	X	X
Team meetings to discuss participant progress and protocol adherence	Weekly	X	X	X	X	X

**Table 2:** Schedule of enrolment, interventions, and assessments for HeLTI Canada fidelity outcomes

				**Study period**								
	**Enrolment**	**Allocation**	**Post-allocation**	**Monitoring frequency**								
**Time point**	*** − t***_***1***_	**0**	***Pregnancy–early childhood***	***Daily***	***Weekly***	***Bi-weekly***	***Monthly***	***Bi-monthly***	***Annually***	***Bi-annually***	***3 months after beginning each phase***	***As needed***
**Enrolment**												
**Eligibility screen**	X											
**Informed consent**	X											
**Randomization**	X											
**Allocation**		X										
**Interventions**												
***Telephone-based collaborative care provided by a HeLTI nurse***			X									
***HeLTI Canada website with individualized webpage and eHealth resources***			X									
**Fidelity assessments**												
***Discussion board***				X								
***Telephone support 24 h***				X								
***Intervention frequency report***						X						
***Intervention logs auditing***								X				
***Intervention group sessions***							X					
***HeLTI Canada App resource bank***											X	
***Participant log-in time in HeLTI Canada App***								X				
***Intervention manual review***										X		
***Nurses exit interview***												X
***Participant satisfaction review***											X	
***Follow-up training***												X
***Healthy conversation skills refresher training***										X		
***Team meetings to discuss participant progress and protocol adherence***					X							

#### Domain 1: Intervention fidelity strategies to monitor study design

Intervention fidelity practices related to study design are intended to ensure that a study can adequately test its hypotheses in relation to its underlying theory and clinical processes [2]. According to the NIH Treatment Fidelity Framework, intervention fidelity goals in this category (see Table 1) include establishing procedures outlined below.

##### Ensure the same intervention dose within conditions

This goal reflects the need to ensure that the intervention ‘dose’ (i.e. number, frequency, and length of contact) is adequately described and is the same for each participant within each arm. A HeLTI Canada intervention manual and a HeLTI Canada nurse training manual have been developed by the HeLTI Canada co-investigators team for use in telephone sessions and will be reviewed on an ongoing basis as needed. Intervention information is outlined in a checklist for each session, as well as in the intervention manual. Included in these manuals are Collaborative Care Scripts which provide scripts for the first contact with a participant, active intervention contact, and continuation intervention contact for each of the four study phases. Intervention activity logs, session checklists, and fidelity checklists have been developed to ensure the intervention is standardized and consistently provided to all trial participants in the intervention group, independent of the HeLTI nurse. Due to the nature of this collaborative care intervention, a fixed length, number, or frequency of intervention cannot be stipulated upfront, given that it is according to the participant’s needs and desires. It is expected to be diverse. However, all information regarding the number, length, and frequency of intervention sessions will be recorded for each, and any deviations from the protocol will be captured. These documents will also be used to ensure intervention fidelity and to determine intervention dosage.

##### Ensure equivalent dose across conditions

This goal reflects the need to ensure that the intervention dose is the same across conditions, such as having an equal number of contacts, the same length of time, and using the same type of information during each contact. The trial coordinator will monitor the intervention sessions via bi-weekly report checks and messages posted on the discussion board that allows the trial coordinator to verify that the intervention is being conducted with the appropriate frequency and provide feedback to the HeLTI nurse who inputted the information. A random 10% sample of all active participant logs is chosen for a bi-monthly audit that provides insight into whether behaviour change techniques are being emphasized by the nurses. At the participant level, the resources available to participants on the HeLTI Canada App are monitored annually to ensure behaviour change techniques are incorporated into the intervention. Adherence to these fidelity measures is also actively monitored through monthly intervention fidelity group sessions between the HeLTI Nurses and the HeLTI Canada team, including the HeLTI Canada lead principal investigator and the lead trainers of the HeLTI Canada intervention.

##### Plan for implementation setbacks

Planning for study design implementation setbacks allows investigators to remedy any deviations from the original study design. In HeLTI Canada, the nurses are the main mechanism by which the intervention is delivered. Therefore, the primary fidelity strategy developed is to hire and train several highly experienced public health nurses to mitigate any potential implementation setbacks that may arise from telehealth coach transition, sick leave, or vacation. Training extra providers beyond those needed ensures availability to re-assign current participants or to assign newly recruited participants avoiding needing to train new providers in a hurry. Additionally, consideration for ongoing hiring of public health nurses will be considered depending on the number of nurses who may exit the study to allow sufficient time to train new nurses. An exit interview will be conducted by the trial coordinator to understand attrition among the HeLTI nurses who leave the trial.

#### Domain 2: Fidelity strategies to monitor provider training

An important area of intervention fidelity is assessing and improving the training of providers to ensure that they have been satisfactorily prepared to deliver the intervention to study participants [2]. The adequacy of training to implement the intervention needs to be evaluated and monitored on an individual basis both during and after the training process. General strategies in this category include standardizing training, measuring skill acquisition by providers, and having procedures in place to prevent drift in skills over time (see Table 3).

**Table 3:** Fidelity of study design strategies and monitoring plan for HeLTI Canada (domain 1)

Goal	Description	HeLTI Canada
Ensure similar dose within the intervention group	Ensure that the intervention ‘dose’ (measured by number, frequency, and length of contact) is adequately described and is similar for each participant	→ Alert for next session with participant built into REDCap, notifying HeLTI nurse → Length, number, and frequency of contact sessions outlined in the intervention protocol → Intervention duration based on the fixed four phases outlined in the intervention protocol → Intervention dose is based on maternal need and is expected to be diverse → Intervention information is outlined in the session checklist and intervention manual → Intervention manual has been developed and will be reviewed 6 months post-intervention initiation → Trial coordinator will monitor the intervention sessions via weekly REDCap checks → Notes section is available at the end of the activity log for HeLTI nurse to document unique information about the session/participant → HeLTI nurse will discuss SMARTER goal achievements at the beginning of each session and monitor progress
Ensure similar dose across phases	Ensure that the dose is the same across the phases, particularly when phases include multiple behavioural targets (e.g. exercise, smoking)	→ Intervention dose is based on maternal need and is expected to be diverse → Intervention phases and corresponding goals are outlined in the intervention manual → Each phase has an ‘active’ (q2 weeks) and ‘continuation’ (q2 months) contact schedule as outlined in the intervention manual → All session details will be tracked in REDCap via an activity log → Trial coordinator will monitor the intervention sessions via bi-weekly REDCap checks → A random 10% sample of all active participant logs is chosen for a bi-monthly audit → At the participant level, the resources available to participants on the HeLTI Canada App are monitored annually
Plan for setbacks	Address possible setbacks in implementation (e.g. intervention providers dropping out)	→ Highly experienced public health nurses to be continuously hired and trained → An exit interview will be conducted by the trial coordinator to understand attrition among the HeLTI nurses who leave the trial

##### Standardized provider training

It is important that training is given to providers in a similar manner to boost provider training fidelity. All HeLTI Canada public health nurses will be trained in Healthy Conversation Skills (HCS) prior to engaging with any participants. The HCS is an engagement strategy based on a social-cognitive model of health behaviour, which emphasizes the role of increasing confidence in promoting behaviour change [10]. It is based on the understanding that providing participants with knowledge alone is insufficient to change behaviour unless they are also motivated and empowered to change. Its focus of Making Every Contact Count is about recognizing opportunities to talk to people about their well-being using the skills of asking and listening. HeLTI nurses will use their skills to explore the barriers that participants in the intervention group have to change their behaviour and ways of overcoming these barriers and setting goals for change. This approach appears more effective than comparable forms of counselling in eliciting behaviour change [11], where even brief interactions can be effective in producing small but important changes in behaviour [12].

In HeLTI Canada, detailed job descriptions are developed to ensure performance criteria can be monitored. All HeLTI Canada nurses are trained in a group setting with a detailed training manual developed and distributed. The manual outlines the study goals, objectives, and design as well as proper intervention delivery and essential skills and training. Training of HeLTI nurses in HCS follows a manual developed by the Medical Research Council (MRC) Lifecourse Epidemiology Unit and consists of a structured 2-day session with supervision for the first 6 months. The same master trainers will be used as much as feasible to ensure the training is provided consistency to all newly hired nurses. Additionally, telephone support from HeLTI Canada Office is available as needed for consultation and support and a HeLTI nurse listserv is available to provide peer support. Training records are kept, ensuring each provider completes all training.

##### Ensure provider skill acquisition

Training provides the foundation for initial skill development and ensuring skill acquisition is necessary to ensure providers provide the intervention as designed. The main skills needed in HeLTI Canada are HCS and the use of evidence-based behaviour change techniques to assist participants with behaviour change. Several strategies are built into HeLTI to solidify skill acquisition including role-playing and walking through potential scenarios that may arise during telephone sessions. Follow-up refresher training will occur after 6 months of intervention initiation and as needed thereafter. Problem-solving sessions and debriefing will occur during follow-up training and via the HeLTI nurse listserv. This portion of the fidelity protocol is monitored via monthly intervention fidelity group sessions and bi-monthly audits of the intervention logs. In the event an audit reveals a lack of skill acquisition, the principal investigator (PI) is notified and can assist coaches with additional training as needed.

The web-based app is also a core element of the intervention. This tool will offer easy access to the eHealth resources recommended by the public health nurse and individualized to the participant’s risks and goals. The resources were selected by a team of co-investigators and experts and evaluated using published validated evaluation tools (i.e. Mobile Application Rating Scale (MARS) or DISCERN checklists) to ensure the quality of the information [13] with feedback obtained from nurses and participants. This process of resource selection and revision will be performed systematically and constantly throughout the full duration of the intervention. In the case of the emergence of new valid tools, we can easily include these tools and make them available to the participants.

##### Minimize ‘drift’ in provider skills

This goal is to ensure that HeLTI Canada nurses do not lose their skills over time. Due to the length and intensity of the intervention (between preconceptions to when the child born in the study is 5 years old), monitoring nurses’ skills over time is critical to ensure the intervention is implemented consistently. HeLTI Canada includes preventive measures to prevent provider drift such as self-monitoring and team collaboration. Nurses are expected to self-monitor their skills using the telephone session checklists described above. The bi-weekly review of the intervention logs also acts as a barrier to drift allowing the PI to assess provider skills throughout the data collection period. Monthly intervention fidelity group sessions occur to discuss intervention issues and reinforce activities. As mentioned above, follow-up training will occur as recommended and required. The HeLTI trial coordinator and programme managers will be available (email, text, telephone) to provide overall support and provide assistance with emergency situations or when safety protocols are triggered. In addition to the written pre-post evaluation of the HCS skills and impact immediately after training, a 1-year follow-up evaluation of how HCS is being implemented in practice is planned.

##### Accommodate provider differences

Because differences such as professional background and previous experience can affect the way study staff implement an intervention, the NIH BCC recommends building measures to ensure provider training is adequate and appropriate based on their individualized level and skill. Within HeLTI Canada, the standardized training previously mentioned allows for the specific skills required for intervention delivery to be honed and accentuated within providers regardless of inherent differences. All HeLTI Canada nurses have received training as mentioned above, and all HeLTI intervention providers are registered nurses with a degree background with a minimum of 5–10 years of public health nursing experience in the province of Ontario or Alberta. Thus, an effort has been taken to minimize intervention-provider differences. HeLTI Canada nurses are also asked to complete a self-report satisfaction questionnaire to understand their comfort level and confidence with intervention implementation.

#### Domain 3: Fidelity strategies to monitor intervention delivery

Intervention fidelity processes that monitor and improve the delivery of the intervention so that it is provided as intended are essential [2]. General goals in this category include using procedures to standardize delivery and checking for protocol adherence (see Table 4).

**Table 4:** Monitoring provider training for HeLTI Canada (domain 2)

Goal	Description	HeLTI Canada
Standardize provider training	Ensure that training is conducted similarly for different providers	→ Detailed job description developed to ensure performance criteria → All HeLTI nurses have been trained in a group setting using a standardized approach using a detailed training manual that has been developed and distributed → Healthy conversation skills training provided by master trainers with structured practices and role-playing included → Telephone support from HeLTI Canada Office is available as needed for consultation and support → HeLTI nurse listserv available to provide peer support
Ensure provider skill acquisition	Train providers to well-defined performance criteria	→ Follow-up training will occur after 3 months of intervention initiation and as needed → Intervention protocol and adherence checklist have been developed with monthly intervention fidelity group sessions and bi-monthly audits of intervention logs → Problem-solving and debriefing will occur during follow-up training and HeLTI nurse listserv → Web-based app which nurses will direct participants to for eHealth resources based on need
Minimize ‘drift’ in provider skills	Ensure that provider skills do not decay over time (e.g. show that provider skills halfway through the intervention period are not significantly different from skills immediately post-training)	→ Monthly HeLTI nurse GoToMeeting will occur to discuss intervention issues and reinforce activities → Bi-weekly review of the intervention logs → Follow-up training will occur as recommended and required → HeLTI trial coordinator and programme manager will be available (email, text, telephone) to provide overall support and provide assistance with emergency situations or when safety protocols are triggered → All participants will complete a ‘satisfaction with HeLTI intervention’ questionnaire 3 months into each new intervention phase. Included in this questionnaire is a therapeutic alliance measure
Accommodate provider differences	Ensure adequate level of training for providers of differing skill levels, experiences, or professional backgrounds	→ All HeLTI nurses have received training and will have a monthly debriefing → All HeLTI intervention providers are registered nurses with a degree background with a minimum of 5–10 years of public health nursing experience in the province of Ontario or Alberta. Effort has been taken to minimize intervention-provider differences

##### Control for provider differences

Differences between nurses have the potential to influence participant behaviour. Work style, credibility, or personality differences may inadvertently affect participant treatment adherence. In order to account for this, all participants will complete a ‘satisfaction with HeLTI Canada intervention’ questionnaire 3 months into each new phase. Included in this questionnaire is a therapeutic alliance measure. Provider differences that may influence treatment delivery can be pointed out and addressed in ongoing staff trainings. Additionally, a participant feedback protocol has been developed. All of this will be accounted for in data analysis.

##### Reduce differences within treatment

To reduce differences within the intervention, a comprehensive training manual with scripts for the first contact with a participant, active intervention contact, and continuation intervention contact for each of the four study phases has been developed and distributed to all HeLTI Canada nurses, as mentioned above. Session checklists have been developed to guide intervention activities and will be monitored through bi-monthly reports by the trial coordinator, as well as the activity logs. Should the activity logs reflect deviation from the intervention protocol, the intervention provider will receive appropriate feedback and additional training if necessary. The discussion board and the monthly intervention fidelity group session will also ensure adherence to these fidelity measures.

##### Ensure adherence to the treatment protocol

Several strategies are in place to ensure participants receive the treatment dose and content as designed. Session checklists have been developed to guide intervention delivery and content (Table 5). Activity call logs will be reviewed for intervention adherence and log completion by the trial coordinator. Call logs ensure that all participants are receiving equivalent number and frequency of calls by tracking the dates and times of scheduled interactions as well as coach adherence to the missed call protocol. The checklists and call logs are audited monthly. Additionally, HeLTI nurses’ notes will be reviewed by the trial coordinator for non-specific intervention effects.

**Table 5:** Monitoring intervention delivery for HeLTI Canada (domain 3)

Goal	Description	HeLTI Canada
Control for provider differences	Monitor and control for participant perceptions of non-specific intervention effects (e.g. perceived warmth and credibility of provider) across the intervention group	→ All participants will complete a ‘satisfaction with HeLTI intervention’ questionnaire 3 months into each new phase. Included in this questionnaire is a therapeutic alliance measure → Participant feedback protocol has been developed
Reduce differences within the intervention	Ensure that providers are delivering the same intervention	→ Session checklists have been developed to guide intervention activities that are standardized → Intervention manual developed and distributed → Activity logs will be monitored in REDCap via reports and review by the trial coordinator monthly → Monthly fidelity group meetings
Ensure adherence to the intervention protocol	Ensure that the intervention is being delivered in the way in which it was conceived with regard to content and dose	→ Session checklists have been developed to guide intervention content → Activity logs will be reviewed for intervention adherence and call log completion by the trial coordinator → HeLTI nurses’ notes will be reviewed by the trial coordinator for non-specific intervention effects
Minimize contamination between the groups	Minimize contamination across intervention/control groups, especially when implemented by same provider	→ The HeLTI Canada Intervention is not available publicly, so the risk of contamination is low → The intervention is telephone based with eHealth resources that are specifically provided to participants via a secure web-based app

##### Minimize contamination between the groups

In terms of minimizing contamination between the groups, the HeLTI Canada intervention is not available publicly, so the risk of contamination is low. The intervention is telephone-based with eHealth resources that are specifically provided to participants via a secure web-based app. Because HeLTI is an individual-level intervention carried out within the digital environment, the chances of participants from different treatment groups interacting are less than in traditional clinic-based interventions.

#### Domain 4: Intervention fidelity strategies to monitor intervention receipt

Receipt of intervention involves monitoring and improving the participant’s ability to not only understand but also act on behaviour changes skills provided during the intervention (Table 6). Participant receipt of treatment is crucial for technology-based studies. Unlike clinic-based behavioural interventions where participants regularly engage in face-to-face contact with providers, telehealth involves minimal in-person contact, and many interactions rely on written or verbal communication where providers may miss non-verbal cues of comprehension. HeLTI Canada will take the following strategies to monitor participant uptake.

**Table 6:** Monitoring receipt of intervention for HeLTI Canada (domain 4)

Goal	Description	HeLTI Canada
Ensure participant comprehension	Ensure that participants understand the information provided in the intervention, especially when they have a low level of literacy/education or are not proficient in English	→ Healthy Conversation Skills underpins the intervention where individualized SMARTER goals are developed by the participant with assistance from the HeLTI Nurse and form the base of intervention content and conversations → SMARTER goals are reviewed at the beginning and end of the session and progress reviewed → If SMARTER goals are not progressing, then barriers are discussed, and goals modified based on participant-identified solutions → Activity logs are completed for each session to guide subsequent sessions
Ensure participant’s ability to use cognitive skills	Make sure that participants are able to use the cognitive skills taught in the intervention (e.g. reframing, problem-solving, preparing for high-risk situations, etc.)	→ HeLTI nurses provide structured sessions at specified time periods to assist participants to achieve their SMARTER goals → HeLTI nurses guide participants on SMARTER goals, monitor progress, and review actual or potential barriers → Exploratory questions guide participants to problem-solve barriers and identify solutions to overcome obstacles to achieving SMARTER goals → Participants rate at the beginning and end of the session how confident they are in achieving their SMARTER goals
Ensure participant’s ability to perform behavioural skills	Make sure that participants are able to use the behavioural skills taught in the intervention (e.g. relaxation techniques, food diaries, cigarette refusal skills)	→ Participant’s ability is continually assessed throughout the duration of the study by regular reviews of nurses’ notes → Participants co-develop their goals with the nurses and rate their confidence in their ability to achieve these goals in each session

##### Ensure participant comprehension

Study outcomes should ideally be interpreted under the assumption that all participants fully understood what was being asked of them during the intervention. Strategies to ensure participant comprehension of treatment protocols increase the likelihood that participant outcomes reflect the effectiveness or ineffectiveness of the treatment, rather than a product of variances in participant comprehension. The HCS approach underpins the HeLTI Canada intervention and is inherently based on using tools to support and empower women and their families to plan for change. This approach recognizes that individuals coming up with their own solution are more likely to take ownership of it. The HCS uses the Making Every Contact Count approach, an evidence-based methodology to improve individuals’ health and well-being by helping them change their behaviour. Rather than telling people what to do, Making Every Contact Count is about recognizing opportunities to talk to people about their well-being using the skills of asking and listening. It is then about empowering people to seek out their own solutions to supporting their own health and well-being. Therefore, at each contact with participant, participant comprehension is assessed and ensure understanding to move forward with their goal as decided on by themselves.

##### Ensure participant’s ability to use cognitive skills

Behavioural interventions such as HeLTI Canada encourage participants to employ cognitive skills such as problem-solving, goal setting, and action planning to meet their goals. The use of HCS enables nurses to gauge participants’ ability to use these cognitive skills through the use of open-ended questions and participant-led goal-setting. HeLTI nurses will provide structured sessions at specified time periods to assist participants to achieve their goals. HeLTI nurses will use exploratory questions to guide the participant to problem-solve barriers and identify solutions to overcome obstacles to achieving goals. Indicators of inadequate cognitive skills include participants setting vague or ambiguous goals, continual failure to meet goals, resistance to goal progression, or ongoing reporting of the same barriers to goal achievement. Participants rate how confident they are in achieving their goals at the beginning and end of the session. Detailed confidential notes taken by nurses after each phone call also serve as a fidelity strategy to ensure participant utilization of these cognitive skills. These narrative notes provide space for nurses to analyse the participant’s progress and are a valuable source of qualitative data that can be used to interpret participant outcomes.

##### Ensure participant’s ability to perform behavioural skills

Several behavioural skills are needed to successfully participate in the HeLTI study. Participants are expected to engage with technology daily, participate in regular exercise, and understand and engage with other activities throughout the study period. The online and telephone-based study design presents challenges to ensuring participant’s ability to perform all behavioural skills since actual observations of participant’s daily behaviour are outside the scope of the study design. Participant ability is continually assessed throughout the duration of the study by regular reviews of nurses’ notes. Additionally, participants co-develop their goals with the nurses and rate their confidence in their ability to achieve these goals in each session.

#### ***Domain 5: Intervention fidelity strategies to monitor enactment of intervention skills (***Table 7***)***

**Table 7:** Monitoring enactment of intervention skills for HeLTI Canada (domain 5)

Goal	Description	HeLTI Canada
Ensure participant’s use of cognitive skills	Ensure that participants actually use the cognitive skills provided in the intervention in appropriate life settings	→ SMARTER goals are reviewed with the participant and progress discussed → Activity logs document all participant interactions with HeLTI nurses → Selected eHealth resources are provided in individualize participant webpage in HeLTI Canada app → Participants rate at the beginning and end of each session how confident they are in achieving their SMARTER goals → Telephone sessions provide an opportunity to discuss the ongoing use of new skills or cognitions underpinning SMARTER goals
Ensure participant’s use of behavioural skills	Ensure that participants actually use the behavioural skills provided in the intervention in appropriate life settings	→ At the beginning of each phase, a detailed risk assessment is completed to determine participant progress in behaviour change and the need for continued or new SMARTER goals → Activity logs are continued from old to new sessions to track change and the use of new behavioural skills → Online questionnaires via REDCap are completed at specified follow-up time points by participants in both intervention and control groups that are linked to phase goals to monitor specific behaviour change and intervention effectiveness → Online measurement of participant login to the online platform and use of the platform

Bellg, A. J., Resnick, B., Minicucci, D. S., Ogedegbe, G., Ernst, D., Borrelli, B.,Czajkowski, S. (2004). Enhancing treatment fidelity in health behaviour change studies: best practices and recommendations from the NIH Behaviour Change Consortium. *Health Psychology*, *23*(5), 443–451. https://doi.org/10.1037/0278-6133.23.5.443

The final domain refers to the performance of learned skills that result in behaviour change in real-life settings to evaluate participants’ actual performance of skills in the intended situations and at the appropriate time [2]. HeLTI Canada will evaluate this domain using the following approaches.

##### Ensure participants’ use of cognitive skills

As previously discussed, cognitive skills such as problem-solving, goal-setting, and action planning are emphasized to help participants meet their goals. The main way adherence to these cognitive skills is assessed is through telephone sessions. Goals are reviewed with the participant, and progression is discussed at each session. Activity logs document all participant interaction with HeLTI nurses and discrepancies between participant’s verbalized intentions to utilize cognitive skills, and their actual use of cognitive skills can be addressed as needed during the coaching sessions. Selected eHealth resources are provided in individualized participant webpages in the HeLTI Canada mobile app and website. The frequency of the calls permits nurses to follow up with participants in a timely manner regarding any new strategies or goals expressed by participants in previous phone calls.

##### Ensure participant’s use of behavioural skills

Similar to monitoring cognitive skills, regular telephone calls are one of the main strategies in place to ensure participants use behavioural skills. At the beginning of each phase, a detailed risk assessment is completed to determine participant progress in behaviour change and the need for continued or new goals. Activity logs are continued from each session to the next to track changes and the use of new behavioural skills. Online questionnaires (conducted through REDCap) are linked to phase goals to monitor specific behaviour change and intervention effectiveness will be completed at specified follow-up time points by participants in both intervention and control groups. In addition to self-monitoring, participants are asked to engage with the technology provided and utilize the behaviour change resources distributed. Periodic checks of participant logins to the online platforms, as well as the time spent in various sections of the platform, provide insight into participant engagement with these resources.

## Discussion

Assessing intervention fidelity is important when determining the effectiveness of technology-based behavioural interventions given the inherent variability within and across interventions. This paper provides an overview of the intervention fidelity protocol for HeLTI Canada, an ongoing randomized controlled trial of a technology-based behavioural intervention delivered by nurses from preconception to when the child born in the trial is 5 years old.

Intervention fidelity monitoring within technology-based interventions should be planned from the outset while acknowledging that fidelity strategies may need to be adapted if they are proving ineffective. Strengths of the current intervention fidelity approach include regular and ongoing monitoring of intervention providers (i.e. HeLTI nurses), using a standardized approach to intervention provision and training using a well-established Healthy Conversation Skills approach, and ongoing tracking of intervention fidelity. For example, one advantage of technology-based interventions is the potential to facilitate fidelity monitoring, which can lead to increased protocol adherence. In HeLTI Canada, participant sessions are tracked as they occur as are all other contacts between the HeLTI nurse and participant allowing for easier review of treatment dose. Furthermore, embedding HeLTI nurse call checklists within the REDCap platform can improve fidelity by ensuring that important content is discussed during each contact.

Possible challenges to intervention fidelity during HeLTI Canada included potential high provider turnover due to the length of the trial and lack of ability to directly monitor behaviour change in real-life settings. Both of these challenges were likely exasperated during the COVID-19 pandemic due to the high burnout and work-related stress among nurses [14] as well as ongoing challenges with in-person assessment. However, a strength of the HeLTI Canada intervention during the COVID-19 pandemic is that the intervention is provided primarily through technology, including telephone calls and web-based intervention, minimizing disruption to the intervention itself.

The benefit of monitoring intervention fidelity during an ongoing behaviour change trial ensures that the intervention is provided consistency throughout, rather than noting intervention drift at the end of the trial. Given that HeLTI Canada is a multi-year study, it is essential to have these strategies in place to ensure that investigators take necessary steps along the way to remedy any deviations from the protocol.

## Trial status

Recruitment began January 2021, and we anticipate recruitment will be completed in 2025.

## Conclusion

Following the NIH BCC recommendation, HeLTI Canada has outlined strategies to monitor and evaluate intervention fidelity related to our technology-based behavioural intervention. Ensuring intervention fidelity requires thoughtful application with effort dedicated to the design of effective strategies that will protect the internal validity of the study. We have presented a protocol for the application of fidelity strategies in HeLTI to improve the scientific findings of our technology-based behaviour change intervention with the goal to evaluate its feasibility, acceptability, and efficacy.

## Supplementary Information

**Additional file 1.** 

## Data Availability

No data is available.

## Abbreviations

BCC: Behaviour Change Consortium
HeLTI: Healthy Life Trajectory Initiative
HCS: Healthy Conversation Skills
MARS: Mobile Application Rating Scale
MCR: Medical Research Council
NCD: Non-communicable diseases
NIH: National Institutes of Health
PI: Principal investigator
REB: Research ethics board
SPIRIT: Standard Protocol Items: Recommendations for Interventional Trials
zBMI: ZBody Mass Index
